# Validation of the Scandinavian guidelines for initial management of minimal, mild and moderate traumatic brain injury in adults

**DOI:** 10.1186/s12916-015-0533-y

**Published:** 2015-12-09

**Authors:** Linda Undén, Olga Calcagnile, Johan Undén, Peter Reinstrup, Jeff Bazarian

**Affiliations:** Lund University, 20185 Lund, Sweden; Department of Pediatric Medicine, Hallands Hospital, 30185 Halmstad, Sweden; Department of Intensive Care and Perioperative Medicine, Lund University, 20502 Malmo, Sweden; Department of Neuroanesthesia, Skane University Hospital and Lund University, 20185 Lund, Sweden; Department of Emergency Medicine, University of Rochester School of Medicine, Rochester, NY USA

**Keywords:** Biomarkers, Brain injury, Computed tomography, Decision rule, Guidelines, Head injury, Management, Mild traumatic brain injury, S100B/S100/S100BB, Traumatic brain injury

## Abstract

**Background:**

Acute management of traumatic brain injury (TBI), in particular mild TBI, focuses on the detection of the 5–7 % who may be harboring potentially life-threatening intracranial hemorrhage (IH) using CT scanning. Guidelines intending to reduce unnecessary head CT scans using available clinical variables to detect those at high IH risk have shown varying results. Recently, the Scandinavian Neurotrauma Committee (SNC) derived a new set of high-IH risk variables for adults with TBI using an evidence-based literature review. Unlike previous guidelines, the SNC guideline incorporates serum values of the brain protein S100B with clinical variables.

**Methods:**

We performed a nested cohort study of adults with mild TBI presenting to six emergency departments in New York and Pennsylvania within 6 h of injury. Patients were managed according to existing guidelines for CT selection. All patients underwent head CT scanning and serum S100B measurement, as well as prospective collection of clinical variables, as a requirement of the parent study. Using the SNC guidelines, S100B values and clinical variables were applied to these subjects, classifying each into one of five pre-defined severity categories, as well as predicting the need for head CT scanning to identify IH. This classification was then compared to actual head CT results to determine guideline sensitivity and specificity.

**Results:**

In total, 662 adults (mean age 42 years, range 18–96; 258 females, 549 Caucasians) were available for analysis; 36 (5 %) had IH on head CT scan. The SNC guidelines had a sensitivity of 97 % (95 % CI, 84–100 %) and a specificity of 34 % (95 % CI, 30–37 %) for the detection of IH on head CT. Application of the SNC guidelines would have resulted in a CT reduction of 32 % (211/662 patients). One patient with low-risk mild TBI and a S100B level under 0.10 μg/L had a traumatic CT abnormality and would have been discharged with strict adherence to the guidelines. However, this patient did not need any intervention for the injury and had a good outcome.

**Conclusion:**

Using the SNC guideline could save approximately one third of CT scans in a pre-selected cohort of mild TBI patients with little or no impact on patient outcome.

## Background

Traumatic brain injury (TBI) is a leading cause of mortality and morbidity [1], and one of the most common reasons to seek emergency department (ED) care [2, 3]. The vast majority of patients with acute (<24 h after injury) TBI are conscious on ED arrival with a Glasgow Come Scale (GCS) of 13–15. These patients are typically defined as mild TBI (mTBI) and constitute approximately 95 % of all TBIs [4]. Although conscious on arrival, a small portion of these patients will have traumatic intracranial findings on computed tomography (CT) and some will require neurosurgical intervention [5]. Many of these are therefore subjected to CT scanning, hospital admission or both. Considering the economic implications of CT scanning and hospital admission, coupled with escalating concerns for radiation risks from CT scans [6, 7], several guidelines and decision rules have been published aiming to guide ED physicians to minimize unnecessary CT scans and/or admission while ensuring a safe triage for mTBI patients [8, 5]. Some of these have been externally validated with varying results [9–11]. Unfortunately, these guidelines are generally not applicable to all mTBI patients presenting in a typical ED. Further, there are concerns that introduction of new guidelines may actually lead to an increase in CT scans [12].

Recently, attention has been focused on efforts using brain-specific biomarkers, mainly protein S100B, in an attempt to reduce unnecessary CT scanning following mTBI [13, 14]. S100B is a dimeric astroglial protein of approximately 21 kD. Although the specific function of the protein has not been established, it seems to have both intracellular and extracellular effects [15]. The half-life of S100B is short, with recent data suggesting a half-life of less than 30 min [16]. Although first thought to be brain specific, studies have shown that low levels of S100B exist in extracerebral tissues and may limit the clinical specificity of S100B in TBI management [17]. Despite this, the high sensitivity and clinical negative predictive value of S100B justifies the use of the protein in TBI management. However, since much of the clinical evidence concerning S100B is relatively recent, it has not been included in clinical guidelines but is nevertheless used clinically in many European countries [18].

**Fig. 1:**
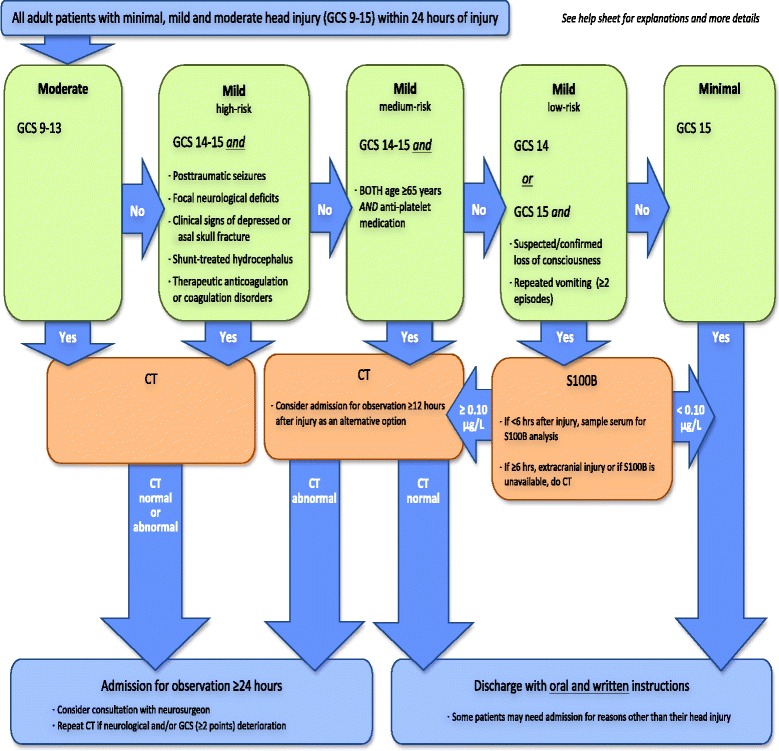
Scandinavian Neurotrauma Committee guidelines [19]

In 2013, the Scandinavian Neurotrauma Committee (SNC) published evidence-based guidelines for initial management of TBI for adults [19] (Fig. 1). These guidelines are designed for patients with acute (<24 h from injury) TBI and for detection of important intracranial injuries, such as those needing neurosurgical intervention and/or intensive care support. The classification of mTBI has further been divided into high, medium and low risk depending on the presence of certain risk factors. The guidelines also include biomarker S100B as a clinical tool for reducing CT scans in a subset of mTBI patients. Although these guidelines were designed for the Scandinavian healthcare systems, validation in an external cohort would be of interest.

## Methods

We performed a retrospective nested cohort study of adults with mTBI presenting to the ED within 6 h of injury. The parent study was a prospective, multicenter, cohort study designed to determine the classification accuracy of serum S100B, serum apolipoprotein A1, and clinical variables for identifying patients with mild TBI and for identifying patients with traumatic abnormalities on head CT [14]. Given the similarities between the variables collected and the variables contained in the SNC, these data permitted an assessment of the performance of the SNC.

Participants were enrolled in the parent study at five hospitals in Upstate New York and one hospital in Pennsylvania between 2008 and 2010. Subjects were eligible for inclusion in the parent study if they were aged 1 year or older, had mTBI as defined by the Centers for Disease Control and Prevention’s National Center for Injury Prevention and Control (a blow to the head or rapid acceleration/deceleration resulting in at least one of the following: a loss of consciousness (LOC) ≤30 min, post-traumatic amnesia ≤24 h, neuropsychological abnormality [any transient period of confusion, disorientation, or impaired consciousness; in children ≤2 years old: irritability, lethargy, or vomiting post-injury], or neurological abnormality [seizure acutely following injury, hemiplegia, or diplopia]) [1]. An additional inclusion criteria was the availability of head CT scanning as part of their clinical care. The Institutional Review Boards for each of the six participating centers approved this study and the process of informed consent. All participants (or guardians of participants) gave informed consent.

### Participants

Subjects were selected from the parent study into this nested cohort if they were adults ≥18 years of age (the SNC guidelines are designed and intended for adults) and had sufficient data present in order to classify patients according to the guidelines.

### Clinically-relevant variables

Subjects participating in the parent cohort were interviewed in the ED by trained research assistants for injury mechanism, initial symptoms, demographics, and medical history. The emergency provider was also interviewed and the emergency chart was reviewed to determine physical exam signs, associated injuries, and GCS score. The decision to collect specific clinical variables was based on their inclusion in two head CT clinical decision rules that were in use at the time the parent study was conducted, namely the New Orleans Criteria (NOC) [8] and the Canadian CT Head Rule [5]. The SNC Head CT guideline, which was published after the parent study was completed, recommended a slightly different set of clinical variables [19]. The subset of clinical variables collected in the parent study that were identical or similar to the variables in the SNC head CT guideline were analyzed in the nested cohort. The extent to which this subset of clinical variables overlap with the variables recommended by the SNC head CT guideline is shown in Table 1.

**Table 1:** Comparison of clinical variables collected and those included in SNC guideline

Clinical variables collected	SNC head CT guideline [19]
Post-traumatic seizure	Post-traumatic seizure
Age	Age ≥65 years
Vomiting, number of times	Vomiting ≥2 times
Glasgow Coma Scale score	Glasgow Coma Scale score
Suspected open skull fracture	Clinical signs of depressed skull fracture
Signs of basilar skull fracture	Clinical signs of basilar skull fracture
Diplopia, paralysis	Focal neurologic deficit
All current neurologic conditions, including hydrocephalus	Shunt-treated hydrocephalus
prothrombin ratio and international normalized ratio, not collected	Coagulation disorders
All current medications including antiplatelet and anticoagulants	Antiplatelet medication
	Therapeutic anticoagulation
Loss of consciousness	Suspected or confirmed loss of consciousness
All extracranial injuries	Significant extracerebral injury
S100B levels	S100B ≤0.10 μg/L

As the variables collected in the parent study were not chosen with the SNC guidelines in mind, certain assumptions were made a priori. As double vision and paralysis were the only neurologically specific symptoms recorded, these were composited to the variable of focal neurological deficit. Further, suspected/confirmed LOC from the guidelines was equated with unsure/confirmed LOC from the cohort data. Significant extracerebral injury was met if internal organ injury, fractures and blast/burn/electrocution injuries were noted. Minor injuries, such as lacerations and bruises, were not classified as significant extracerebral injuries.

### Head CT scans

At each study site, head CT scans were interpreted by board-certified radiologists who were blinded to the laboratory results. The final reading entered into the radiology image database at each institution was used to determine the presence or absence of intracranial abnormalities. Traumatic CT abnormalities were defined as subdural hematomas, epidural hematomas, subarachnoid hemorrhage, edema, skull fracture, and cerebral contusions.

### Blood draw and sample handling

Blood for S100B sampling was drawn from mTBI subjects within 6 h of the time of injury. Four milliliters of whole blood was drawn into a serum separator tube and immediately placed on ice. Within 60 min, the blood was centrifuged at 3000 rpms for 10 min and the serum was aliquoted into 500 μL tubes frozen at −80 °C.

### S100B assay

Serum S100B concentrations were determined by a fully automatic electrochemoluminometric immunoassay (Elecsys S100; Roche Diagnostics, Penzberg, Germany) with a detection limit of 0.005–39 lg/L. The analyte was sandwiched between two monoclonal antibodies directed against the beta-chain of the S100 dimer. Then, streptavidin-coated microparticles were added and the immunocomplex was bound to the solid phase. In the measurement cell, unbound components were removed and a defined voltage used to initiate the electrochemiluminescent reaction. The resultant light emission was then measured using a photomultiplier. S100B assays were performed from November to December 2010. Resulting S100B values were not available to the emergency physicians caring for the subjects involved in this study, nor where they available to interviewers and trained research assistants. Thus, providers and research personnel were blinded to S100B results.

### Outcome

One month after the initial ED visit, outcome was determined by telephone interview using the Rivermead Post Concussion Questionnaire [20, 21]. Subjects were asked to rate the severity of 16 post-concussive symptoms (such as headache), compared to pre-injury, on a Likert scale ranging from “0” (absent) to “4” (severe). Total scores thus ranged from 0–64. The interviewer was blinded to the details of the ED visit.

### Analysis

Using the SNC guidelines, S100B values and clinical variables were used to classify each subject into one of the five SNC-defined head injury severity categories (moderate TBI, mTBI/high risk, mTBI/medium risk, mTBI/low risk, and minimal TBI). In order to estimate the ability of the SNC to determine head injury severity, the prevalence of traumatic CT abnormalities in each severity category was calculated and compared. Because the number of CT+ subjects was ≤5 in two severity groups (moderate-risk and medium-risk mTBI), the Fishers exact test, rather than the χ^2^ test, was used to make these comparisons. Given the fixed samples sizes of each SNC severity group and the number of CT+ subjects in each group, the power to detect the observed differences in CT+ prevalence between groups – assuming a Type 1 error rate of 0.05 – ranged from 0.132 to 0.717. The need for head CT scanning as predicted by the SNC was then compared to actual head CT results to determine guideline sensitivity and specificity.

## Results

**Fig. 2:**
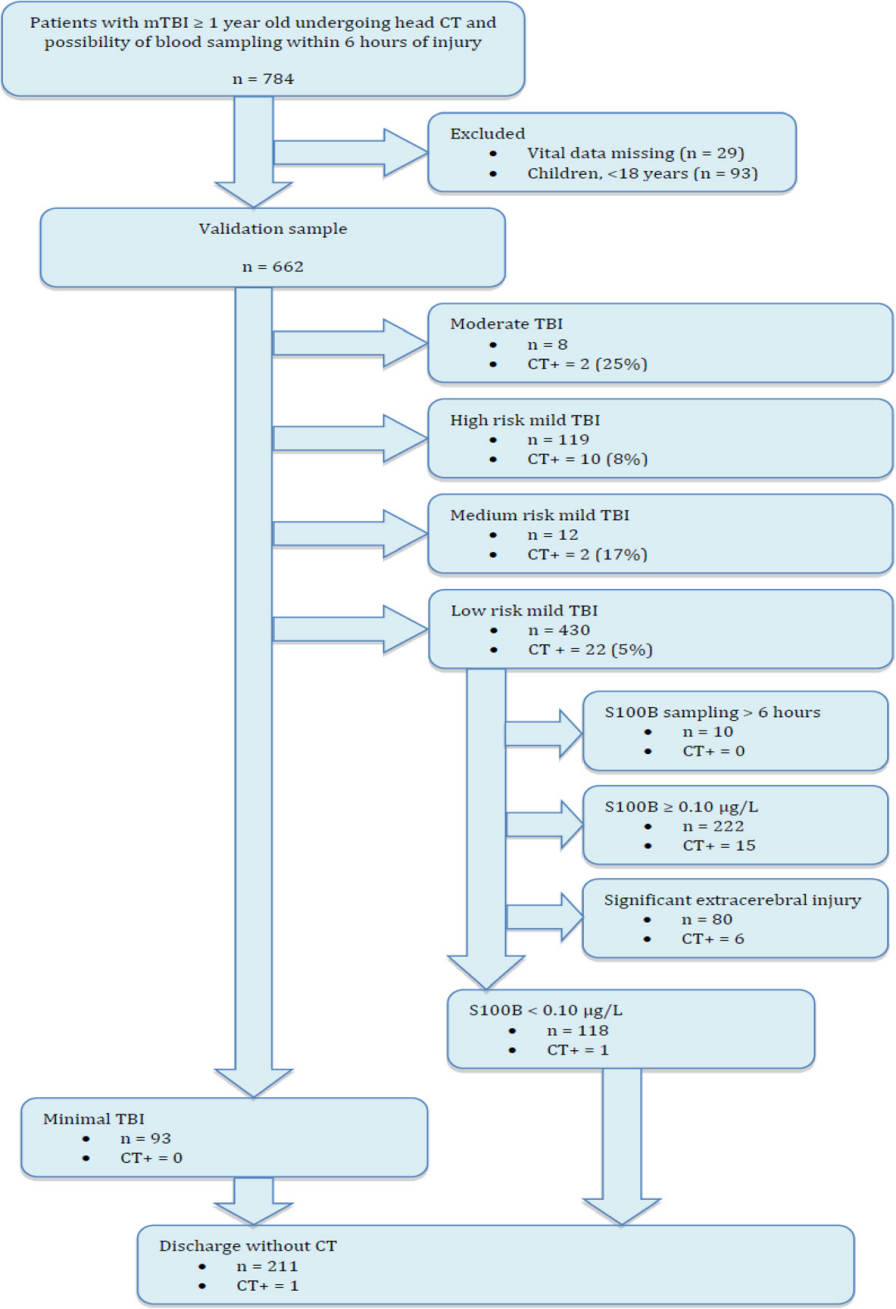
Study flow chart

During the study period, 784 subjects with mTBI were enrolled into the parent study; 93 were children and therefore not considered for the guidelines. In 29 patients, vital data was missing (mainly GCS scores), which made it impossible to accurately classify the patients and they were therefore excluded. Thus, 662 patients were eligible for analysis (Fig. 2). Most subjects in the nested cohort were Caucasian and male (Table 2).

**Table 2:** Characteristics of the nested cohort

Variable	
Age, mean (range)	42 (18–96) years
Sex, percentage female	39 %
Race	83 % Caucasian
Head CT results	37 CT+ (6 %)

### SNC guidelines and head injury severity

CT scans were positive (CT+) for traumatic abnormalities in 36/662 patients (5 %). Eight patients showed cerebral contusions, six had traumatic subarachnoid hemorrhage, four had subdural hematomas, two had petechial hemorrhage/shear injury, one had cerebral edema, one had a linear skull fracture, and one had an epidural hematoma. The remaining 13 patients had a combination of intracranial traumatic abnormalities. No patients in the cohort needed neurosurgical intervention and none died as a result of the TBI.

**Fig. 3:**
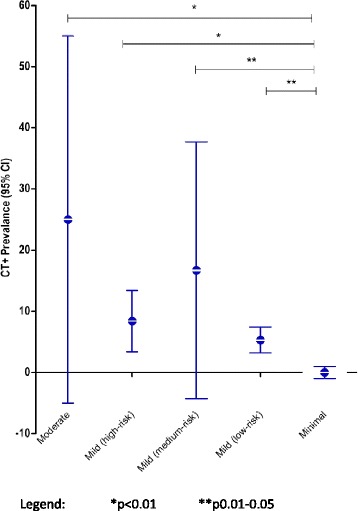
Prevalence of traumatic CT abnormalities by SNC guideline severity categories. *P <0.01, **P = 0.01–0.05

Eight subjects were classified by SNC as moderate TBI, 119 as high-risk mTBI, 12 as medium-risk mTBI, 430 as low-risk mTBI, and 93 as minimal TBI (Table 3). The prevalence of CT+ was highest in the moderate TBI group (25 %) and lowest in the minimal TBI group (0 %). Compared to the minimal TBI group, the CT+ prevalence was significantly higher in the moderate TBI (*P* = 0.006), in the high-risk mTBI group (*P* = 0.003), in the medium-risk mTBI group (*P* = 0.012), and in low-risk mTBI group (*P* = 0.021; Fig. 3). The CT+ prevalence in the moderate TBI group (25 %) was higher than the low-risk mTBI group (5 %), but this difference did not reach statistical significance. The CT+ prevalence in the medium-risk mTBI (17 %) was higher than that of the high-risk mTBI (8 %), but this difference did not reach statistical significance.

**Table 3:** Performance of SNC guideline in validation cohort

		CT results		
		+	−	Total
SNC guideline	CT	35	416	451
	No CT	1	210	211
	Total	36	626	662

Overall, a 32 % reduction in CT scanning was observed if SNC guidelines were used; 1 missed patient (low-risk mild with S100B ≤0.10 μg/L), see text for details; Prevalence of CT findings: 5 %; Sensitivity: 97 % (95 % CI, 84–100 %); Specificity: 34 % (95 % CI, 30–37 %); Negative predictive value, 100 % (95 % CI, 97–100 %); Positive predictive value, 8 % (95 % CI, 6–11 %)

### SNC guidelines and prediction of traumatic abnormalities on head CT

The SNC guidelines classified 451 subjects as needing a head CT scan, and 211 as not needing one. The SNC guidelines had a sensitivity of 97 % (95 % CI, 84–100 %) and a specificity of 34 % (95 % CI, 30–37 %) for predicting traumatic CT abnormalities (Table 3).

In patients with high-risk mTBI, 84 patients exhibited double vision as a risk factor and four of these had CT+ findings. Of 19 patients with paralysis, none had a CT+ lesion. Of 18 patients with seizures and 12 patients with clinical suspicion of open/depressed skull fracture, two cases in each showed CT+ findings. Finally, eight patients with anticoagulant use and six patients with clinical signs of basal skull fractures each showed one case of CT+. Overall, 430 patients were classed as low-risk mTBI (65 % of the total sample). Of these, 340 were eligible for S100B sampling according to the SNC guidelines (10 underwent sampling more than 6 h after injury and 80 patients had significant extracerebral injuries). Of these, 118 had levels <0.10 μg/L and 222 had levels ≥0.10 μg/L (35 % below cut-off). In patients with extracerebral injury, six had CT+ and all of these had elevated S100B levels. None of the 10 patients with sampling done after 6 h from injury had CT+ results.

**Fig. 4:**
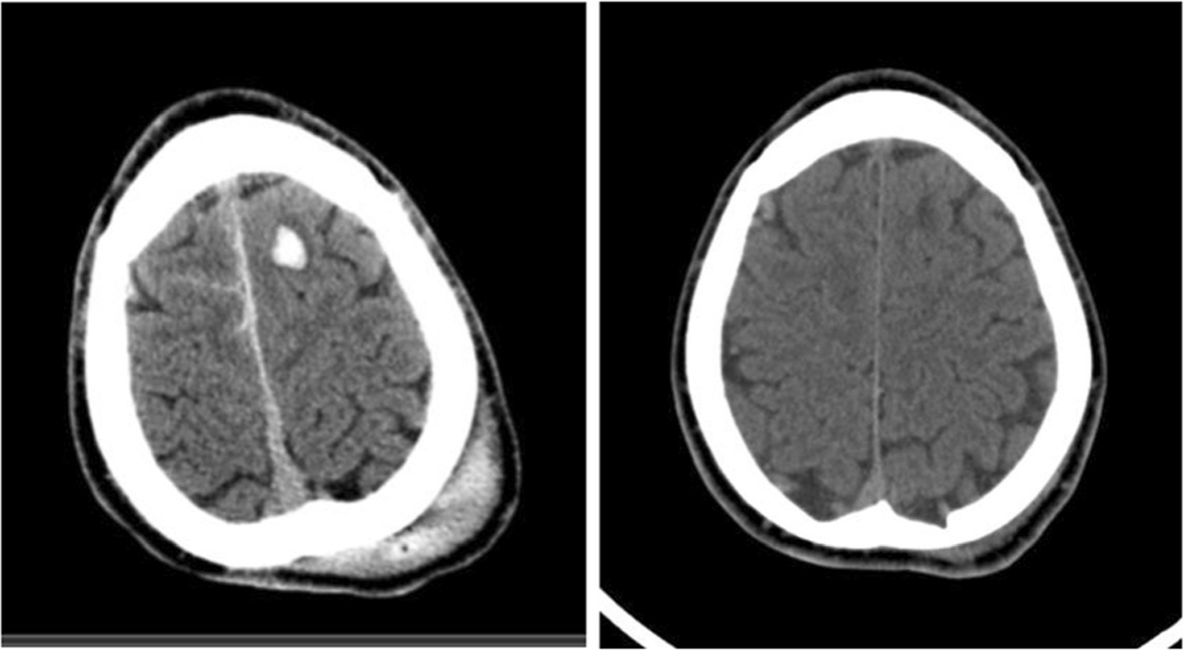
False negative subject. A 20-year-old with GCS 14 and unclear episode of loss of consciousness. The patient would be classed as low-risk mild traumatic brain injury and had a S100B of 0.09 μg/L. CT shows a small contusion and extracranial soft tissue swelling. The contusion subsided on follow-up CT after 25 days. The patient did not need any intervention or treatment and was discharged from the inpatient unit with a good neurological outcome

In total, application of the SNC guidelines to this validation sample would have resulted in a CT reduction of 32 % (211/662 patients); one patient with a low-risk mild TBI and a S100B level under 0.10 μg/L had a traumatic CT abnormality. This patient was a 20-year-old male presenting at the ED after a motor vehicle accident (without ejection) with a GCS of 14 and LOC (unclear time period). CT showed a small cerebral contusion which subsided on follow-up CT scans (Fig. 4). He was discharged home from the inpatient unit without needing medical or surgical intervention for his injury and had a good neurological outcome on follow-up. His total Rivermead Post Concussion Questionnaire score was low (11 out of 64) and he had new symptoms of moderate fatigue and mild issues of frustration and poor memory.

## Discussion

In response to escalating healthcare costs, care providers have a responsibility to manage patients within health-economic considerations [22]. TBI, in particular mTBI, represents a significant burden for hospitals and ED facilities in developed countries. Existing guidelines for management of such patients differ in sensitivity and specificity with respect to detection of CT findings, traumatic CT findings, clinically important CT findings and need for neurosurgical/intensive care intervention [9, 11]. The SNC guideline offers a comprehensive aid to management of all adults with TBI and includes CT management options.

The results indicate that the SNC guidelines seem to predict TBI severity within a cohort of patients with GCS 13–15. The CT+ prevalence in the minimal TBI group was significantly lower than each of the other four severity groups. In addition, the CT+ prevalence in the moderate TBI group was five times higher than that of the low-risk mTBI group, but this difference was not statistically significant, likely due to the small number of subjects (n = 8) in the moderate risk group. Counterintuitively, the CT+ prevalence in the medium-risk mTBI group was over twice that of the high-risk mTBI group, but this difference was also not statistically significant, likely due to the small number of subjects (n = 12) in the medium-risk mTBI group.

Adherence to the SNC guidelines would have resulted in a 32 % reduction in CT scans in the present population – a population clinically judged to need a CT scan according to local routines. Since many of these patients were also admitted to hospital, the cost saving potential is substantial. However, one patient, who was classified by the SNC guideline as low-risk mTBI and had a low S100B level (0.09 μg/L, just below cut-off), showed a traumatic abnormality on CT scanning. The finding was relatively minor (small contusion) and did not need any specific treatment. Missing this CT abnormality would therefore not have had an impact on the care and outcome of this patient. S100B is not 100 % sensitive and less significant, non-neurosurgical lesions, such as the lesion in this study, may be missed using the present cut-off [23, 24]. Additionally, the SNC guidelines were designed to primarily detect patients needing neurosurgical or other specific intervention, with traumatic CT abnormalities being a secondary, yet important, goal [19]. It is likely that the difference in medico-legal attitudes between countries may influence the view on this matter. In Scandinavia, missing uncomplicated intracranial complications that do not need specific intervention in patients with good outcome is acceptable, especially if this implies resource saving. However, this may not be true for other countries such as the United States and Canada.

Since S100B is currently unavailable in the US, the management according to the SNC guidelines would have differed in that patients with low-risk mTBI would have had a CT scan. In this scenario, no patients would have been missed but the CT use would have been reduced to 14 % (93/662).

S100B is included as an option in the guidelines for those centers with the ability to perform 24/7 real-time analysis. As most Scandinavian centers have this possibility, S100B is now widely used in this setting. Published reports of S100B in clinical use [18] and unpublished summaries of current use with the new guidelines have shown very promising results. However, these recent observations need to be scientifically examined, a process which is currently underway via a validation study in Scandinavia. Further, the practicality of using the SNC guidelines would be of interest but could not be examined in the present study. A guideline would have to be practically viable for the treating of professionals in order for such a tool to be clinically useful.

The sensitivity and specificity figures reported here are not reflective of an unselected cohort of TBI patients, but rather a selection of patients where current guidelines advocate a CT scan. In a more unselected cohort, the sensitivity and negative predictive ability would reasonably be higher as the present study had already selected a population with a higher risk for CT abnormalities (higher pre-test probability). Therefore, it is also difficult to compare the performance of different guidelines as the cohort is pre-selected. The NOC criteria, for example, would reasonably advocate CT scans on many patients not considered for inclusion into this cohort and can only be used on the subset of patients with GCS 15. Additionally, both the NOC and Canadian CT Head Rule criteria can only be applied to patients with specific symptoms (LOC and LOC, amnesia or confusion, respectively), unlike the SNC guidelines, which are designed for all adult patients following a non-severe TBI. The only correct method of comparing these guidelines would be in an unselected series of all TBI patients presenting at the ED.

### Limitations

The S100B cutoff used in the SNC guidelines was derived from studies involving mostly Caucasian populations. This cutoff might not perform the same in subjects of color, such as some of those in the current study. However, this would rather affect the specificity of S100B (i.e. more false positives) and therefore theoretically reduce the CT saving ability of S100B in non-Caucasian populations. S100B as a single test had a 35 % CT reduction ability in this study, which is similar to other cohorts [25].

Although the reported cohort is relatively large, the absolute number of CT+ patients was small and no patients needed neurosurgical or specific medical intervention. A much larger cohort would be necessary to fully examine these aspects. Further, although the original patient inclusion was prospective, the validation of the SNC guidelines was retrospective. A purpose-designed prospective study is recommended and currently underway.

## Conclusion

The updated SNC guidelines can accurately classify injury severity and may further reduce CT scans in a selected population of patients with TBI requiring CT scanning. The one patient missed by the guidelines did not require any intervention and had a good outcome.

## Availability of supporting data

Raw data is currently not available to publicly share as further analysis of the parent cohort is planned. However, the authors (specifically JB) will consider sharing data upon personal request.

## Abbreviations

CT: Computed tomography
ED: Emergency department
GCS: Glasgow Come Scale
LOC: Loss of consciousness
mTBI: Mild TBI
NOC: New Orleans Criteria
SNC: Scandinavian Neurotrauma Committee
TBI: Traumatic brain injury
